# Immersive and desktop virtual reality in virtual fashion stores: a comparison between shopping experiences

**DOI:** 10.1007/s10055-023-00806-y

**Published:** 2023-05-16

**Authors:** Marina Ricci, Alessandro Evangelista, Annalisa Di Roma, Michele Fiorentino

**Affiliations:** 1 Department of Mechanics, Mathematics, and Management, Polytechnic University of Bari, Via Orabona, 4, Bari, Italy; 2 Department of Architecture, Construction and Design, Polytechnic University of Bari, Via Orabona, 4, Bari, Italy

**Keywords:** Virtual reality, Retailing, Fashion industry, Shopping experience, User study

## Abstract

With the high growth and prosperity of e-commerce, the retail industry needs to explore new technologies that improve digital shopping experiences. In the current technological scenario, Virtual Reality (VR) emerges as a tool and an opportunity for enhancing shopping activities, especially for the fashion industry. This study explores whether using Immersive Virtual Reality (IVR) technologies enhances the shopping experience in the fashion industry compared to Desktop Virtual Reality (DVR). A within-subject experiment was carried out involving a sample of 60 participants who completed a simulated shopping experience. In the first mode (DVR), a desktop computer setup was used to test the shopping experience using a mouse and keyboard for navigation. The second mode (IVR) exploited a Head-Mounted Display (HMD), and controllers, that allowed navigation while seated on a workstation to avoid sickness. Participants had to find a bag in the virtual shop and explore its features until they were ready to purchase it. Post-hoc measures of time duration of the shopping experience, hedonic and utilitarian values, user experience, and cognitive load were compared. Results showed that participants experienced higher hedonism and utilitarianism in the IVR shop compared to DVR. The cognitive load was comparable in both modes, while user experience was higher in IVR. In addition, the time duration of the shopping experience was higher in IVR, where users stayed immersed and enjoyed it for longer. This study has implications for fashion industry research, as the use of IVR can potentially lead to novel shopping patterns by enhancing the shopping experience.

**Supplementary Information:**

The online version contains supplementary material available at 10.1007/s10055-023-00806-y.

## Introduction

Nowadays, we are witnessing exponential growth in the use of e-commerce in the retail industry (AWS for Industries 2021), and the Covid-19 crisis has accelerated this digitization process by several years (McKinsey 2020; Shankar et al. 2021). This phenomenon also drives the demand for retail technologies such as Internet of Things (IoT), Artificial Intelligence (AI), and Virtual and Augmented Reality (VR, AR) that improve the user’s shopping experience (AWS for Industries 2021).

In this framework, VR technologies represent an e-commerce opportunity (Grewal et al. 2018) to produce satisfying consumer experiences similar to those experienced in physical stores (Alcañiz et al. 2019) by enriching online consumer experiences in the emerging Metaverse (Shen et al. 2021).

By definition, VR concerns “the use of computer simulation that enables interaction with a virtual, three-dimensional, visual environment through digital representation” (Biocca 1992). Users are usually immersed in the digital environment through a Head-Mounted Display (HMD), but they do not physically share the same space with the objects or environment reconstructed through VR (Sheridan 1992).

It is forecasted that most Internet users worldwide will use VR headsets on a daily basis within the next 7–10 years (Rosedale 2017). In fact, VR applications are “rapidly evolving and increasingly used in retail environments” (Javornik 2016; McCormick et al. 2014).

The global VR market was worth USD 21.83 billion in 2021 and is predicted to grow at a compound annual growth rate (CAGR) of 15.0% between 2022 and 2030 (Grand View Research 2020). These data show that consumers are ready to embrace immersive technologies in their daily lives (Rosedale 2017).

In today's global economy, fashion is one retail sector that could benefit from VR. Moreover, the fashion industry is growing at a very fast pace, with a predicted CAGR of 11.45%, resulting in a market volume of US $1.37tn by 2025 (Statista Market Forecast 2022).

The use of retail technology in the fashion industry plays a key role in enhancing the user experience. Indeed, VR represents one of the most interesting candidates for next-generation e-commerce and could give brands the opportunity to improve the shopping experience (Morotti et al. 2020; Park et al. 2018). In fact, VR can preserve existing web-based services while helping reduce the mistrust of the most demanding online users by increasing the digital informativeness of clothes and accessories through 3D models and interactivity.

Despite the growth of e-commerce, several aspects need to be improved with online shops for the fashion industry. For example, current online shopping systems show products only through text and photos and cannot provide end users with an interesting shopping experience (Wu et al. 2019). These modes of product presentation fail to convey product features to users clearly. Furthermore, unnatural interaction techniques, such as scrolling through a list or navigating through product information pages, raise consumers' cognitive load (e.g., frustration) (Wu et al. 2019) and, as a result, negatively impact their shopping experience (e. g., presence, immersion, and attractiveness) (Peukert et al. 2019).

In contrast, Immersive VR (IVR) can generate several potential advantages, particularly for fashion retail. Indeed, IVR allows the configuration of products at 360°, showing users the configured product through an immersive 3D visualization. Thus, allowing the user to understand better the configured product's features that could be difficult to perceive through a flat 2D image shown on a traditional monitor (Ricci et al. 2023). In fact, by exploiting IVR, end-users can view products from different perspectives and showcase the details of items (e.g., show the material and texture) (Wu et al. 2019). This condition is amplified for high-quality products that feature distinctive shapes, materials, and finishes and require great purchase confidence due to their cost (Fiorentino et al. 2022). For example, buying an expensive bag can be considered an emotional process that requires an accurate representation of the 3D product.

The “virtual” component of the experience can be integrated into e-commerce in both immersive and non-immersive ways (Ricci 2022). This paper investigates how different display and interaction systems in the virtual environment can influence the shopping experience of the product. Therefore, we compare a shopping experience on a desktop computer – Desktop Virtual Reality (DVR) – and a shopping experience in IVR, by assessing the measures of time duration of the shopping experience, hedonic and utilitarian values, cognitive load, and user experience.

By definition, the hedonic shopping value reflects the value gained through the multisensory, imaginative, and emotional aspects of the shopping experience. In contrast, the utilitarian shopping value reflects the efficient acquisition of products and/or information and can be seen as a more task-oriented, cognitive, and non-emotional shopping outcome (Babin et al. 1994). The cognitive load, on the one hand, refers to the perceived cognitive load related to the task to be performed in the shop (Hart and Staveland 1988). On the other hand, the user experience concerns the level of attractiveness, perspicuity, efficiency, dependency, stimulation, and novelty of the overall shopping experience (Laugwitz et al. 2008).

Then, a comparative study is carried out between an IVR shopping experience and a DVR shopping experience. To the best of our knowledge, there is a lack of literature comparing immersive and non-immersive shopping experiences in the fashion retail field. Thus, in this work, we want to address the following Research Questions (RQs):

o RQ1—Is the time duration of the shopping experience longer in IVR than in DVR?
p RQ2—Can the proposed IVR-based shopping experience deliver higher rates in terms of hedonic and utilitarian values than a DVR-based?
q RQ3—Can the proposed IVR-based shopping experience present a cognitive load comparable to the DVR-based?
r RQ4—Can the proposed IVR-based shopping experience improve the user experience compared to DVR-based?

The remainder of this paper is structured into five sections. The first describes the state-of-the-art of VR technologies applied to the shopping experience, focusing on comparative studies in retail. The second describes the methodology to carry out the comparative study. The third describes the results in terms of subjective and objective measures. The fourth presents a discussion of the results. Lastly, we report our conclusions and future works.

## Related work

Although VR has proven its effectiveness in the field of fashion retail, the scientific literature is scattered and still presents limited studies (Xi and Hamari 2021). Furthermore, only a few studies present experimental designs. For this reason, further research is needed to determine how VR technology can improve the user’s shopping experience.

Lau et al. (2014) discussed the design of interactivity for enhancing consumers' shopping experiences. They created a VR shop and interviewed a sample of 61 participants. The virtual environment allowed the participants to browse, explore, and interact with products for 15 min. The interview revealed that the participants engaged themselves and enjoyed the experience demonstrating how interactive design could enhance consumers’ shopping experiences (Lau et al. 2014).

Moes and Van Vliet (2017) explored how customers can experience shopping in a fashion store without actually being there by using visual content. The study investigated the effects of viewing a photo, a 360-degree photo, and a VR photo of a physical store. Two experiments were conducted to address the research questions with between-subjects designs. The independent variable was the form of communication while the dependent variables were the real shop experience, grade, holistic shop experience, visitor intention, purchase intention, opinion of a physical shop, and recall. Consumers who viewed the VR image of the store had a more pleasant shopping experience, a higher purchase intention, and a higher intention to visit the shop than customers who had only seen the regular photo or the 360° photo of the shop (Moes and Vliet 2017).

The effects of social circumstances on users' perceptions of the virtual body and their emotional and psychological states were examined by Dzardanova et al. (2017). They carried out a user study while immersing a sample of 54 participants in a VR apparel store. Users alone or with a virtual salesman observed their avatar's naked virtual body. Results showed that the presence of a second character did not affect degrees of body ownership illusion or presence, but caused a significant emotional reaction, demonstrating that social context and social presence have an impact on users (Dzardanova et al. 2017).

Donatiello et al. (2018) developed the "Fashion Island" application, a concept of a virtual fitting room in VR where users can dress avatars by pointing and clicking on clothing and accessories. The user could choose which types of clothing or accessories they want using a basic graphical interface. A group of 13 volunteers participated in the experiment and provided opinions on the interface's usability, overall experience, and cybersickness. Results were generally encouraging, paving the way for future studies (Donatiello et al. 2018).

Park et al. (2018) investigated the user experience in virtual stores and how it affects shopping outcomes. To this end, they designed a VR store for female customers, recruiting 40 women for the experiment. Participants were asked to enter and explore the store freely during a one-hour session. In addition, a questionnaire was administered to assess telepresence, perceived realism, pleasure, arousal, attitude, purchase intention, and simulator sickness. According to preliminary findings, significant purchasing outcomes such as pleasure, attitude toward virtual stores, and purchase intention were favorably associated with the IVR experience. This indicates that using VR as a new shopping tool can improve the engagement and experience of customers (Park et al. 2018).

Jang et al. (2019) examined the roles of vividness and interactivity in customers' approach intentions toward an IVR store. A sample of 101 users tried out the VR store with an HMD. The findings demonstrated that participants' perceptions of higher vividness and interactivity are related to stronger approach intentions and that these positive benefits were successively moderated by participants' perceptions of telepresence and experiential shopping value (Jang et al. 2019).

Lau and Lee (2018) focused on consumers' shopping experiences in StereoVR, by designing “FutureShop” and evaluating its viability in enhancing customer contact compared to internet purchasing. A sample of 59 participants answered a questionnaire after spending 30 min in FutureShop in order to measure consumers' purchase intention, interactive shopping, and hedonic user experience. The findings suggest that VR could improve hedonic value, interactive retail experiences, and purchase intention (Lau and Lee 2018).

Morotti et al. (2020) explored the advantages of speaking and verbally communicating with a VR assistant representing a salesman in order to discuss the potential of employing vocal instructions in a VR fashion store. They designed a survey using the Technology Acceptance Model technique to assess the perceived ease of use and the perceived usefulness of the voice-enabled interface. Only nine fashion experts tested the application. Preliminary findings indicated that VR could provide effective experiences and the integration of the voice assistant may help to simplify and naturalize the virtual experience (Morotti et al. 2020).

The contributions span different areas, from computer science to marketing and management contexts, proving the interdisciplinary nature of academic research on VR in the context of fashion retail (Bonetti et al. 2017). However, the intention to enhance the shopping experience by exploiting VR technologies emerges from all the contributions.

### Comparative studies in retail

In the literature, there are few comparative studies assessing the shopping experience. Three possible scenarios were reported for comparison: real shop, IVR shop, and DVR shop.

Bressoud (2013) tested a new adult cereal with 200 customers in France to compare the differences between a real and a virtual shopping experience. The findings indicate that all attitudinal metrics are comparable in terms of cognition and conation, while affect and behavior cannot be compared across the two methodologies. In conclusion, early-stage testing of novel concepts can be done using virtual stores, but they should not be used as a basis for decisions on new product launches (Bressoud 2013).

Waterlander et al. (2015) designed and validated a virtual supermarket by comparing virtual and actual food shopping behavior. They used the Presence Questionnaire Items Stems to gather participant input on the perceived sense of presence. In New Zealand, a sample of 123 eligible main household shoppers was required to conduct three shopping occasions in the virtual supermarket over three consecutive weeks. The four food categories with the highest relative prices were the same in both the virtual and actual supermarkets (i.e., fresh fruit and vegetables, bread and pastries, lentils, and meat and fish). According to the findings, real and virtual grocery shopping patterns are similar. Overall, the virtual supermarket is a reliable method for analyzing consumer food-buying patterns (Waterlander et al. 2015).

Van Herpen et al. (2016) compared VR to a 2D graphical representation of the same retail environment while maintaining the same store assortment, display, and product information. The two laboratory conditions were then compared to a real store. A sample of 90 students was randomly allocated to one of three groups: (a) a simulated shelf display, (b) a VR shelf display, or (c) a shelf display picture. The shelf display included 16 distinct red wines with short descriptions and prices. The study presented preliminary evidence for the benefits of integrating VR rather than pictures in consumer behavior research. Also, the findings suggested that VR could encourage more habitual purchasing processes and ensure consistent responses to display attributes (van Herpen et al. 2016).

Peukert et al. (2019) built and experimentally tested a theoretical model that explains how immersion impacts adoption in a shopping environment. To this aim, they designed a virtual shelf containing various types of muesli, which participants experienced by wearing an HMD or viewing product models in 3D on a desktop. They discovered that immersion does not affect consumers' intention to return to the shopping environment. However, extremely immersive retail environments positively affect a hedonic path through telepresence while, surprisingly, negatively influencing a utilitarian path through product diagnosticity (Peukert et al. 2019).

Pizzi et al. (2019) introduced a theoretical model for explaining consumer in-store reactions based on channel and shopping orientation. The concept was tested in the context of a large European grocery retail chain by replicating the same shelf layout of a target category (i.e., industrial confectionery) in both a real and a VR store. They used a quasi-experimental between-subjects experiment to assess hedonism, utilitarianism, store satisfaction, and perceived assortment size. Participants interacted with the same shelf both in the real and VR store. According to the findings, VR negatively impacts satisfaction regulated by perceived assortment size and stimulates utilitarianism and hedonism. After the VR experience, customers reported high levels of all tested outcome variables (Pizzi et al. 2019).

Schnack et al. (2019) investigated if using VR technology in a virtual simulated store improves perceived telepresence and usability over traditional PC technology. They conducted two experiments (VR group; desktop group) with a between-subject design and a sample of 111 participants completed a simulated shopping trip. Participants purchased grocery items in each environment and post-hoc measures of perceived telepresence and usability ratings were compared. The results showed that participants in the VR group experienced a greater feeling of immersion and perceived naturalness in their interactions with the store environment than the desktop group (Schnack et al. 2019).

Lombart et al. (2020) explored the effects of a real shop, a non-immersive virtual store, and an immersive virtual store on consumer perceptions and purchasing behavior toward Fruits and Vegetables (FaVs). They conducted a between-subject experiment with a sample of 192 business school students to achieve the study objective. According to the findings, consumers' impressions of FaVs in both non-immersive and immersive virtual stores were comparable to those in real stores. When compared to a real store, people purchase more FaVs in both non-immersive and IVR settings. The results also revealed that when evaluating the FaVs in IVR, customers rely more on extrinsic cues (i.e., prices) and less on intrinsic cues (e.g., appearance) than they do in the real store (Lombart et al. 2020).

Although all contributions share the same retail product category (i.e., grocery), some authors suggest further studies for high-involvement categories such as fashion products (e. g., clothes, accessories), expecting hedonic and utilitarian values to be more pronounced and positive for these product categories (Peukert et al. 2019; Pizzi et al. 2019; Scarpi 2006). The results of this analysis are consistent with other researchers’ findings reporting that hedonic values provided by interactive technologies result in stronger purchase intentions than passive product presentations in traditional Web-based shopping practices (Lau and Lee 2018) (Table 1).

**Table 1:** Summary of prior literature about comparative studies

Study	Comparison	Retail product category	Dependent variables
Bressoud (2013)	VR vs. experimental real store	Grocery (Muesli)	Affective attitude (Wahlers et al. 1986), Cognitive Attitude (Filser 1994), Conative Attitude (Holbrook and Hirschman 1982); Time of the experience; Purchase rate
Waterlander et al. (2015)	VR vs. experimental real store	Grocery (a: fresh fruit and vegetables, b: bread and bakery, c: dairy, d: meat and fish)	Presence (Witmer and Singer 1998); % Expenditures; % Items purchased
Van Herpen et al. (2016)	Real vs VR vs Picture	Grocery (a: Fruit and vegetables; b: milk, c: biscuits)	Presence (Witmer et al. 2005; Witmer and Singer 1998), Number of products selected, Level of variety seeking, Purchase of store brands/generics, Purchase of national brand, Amount of money spent, Purchase from top/middle/bottom shelves, Purchase from left / middle / right shelves
Peukert et al. (2019)	VR vs DVR	Grocery (Muesli)	Hedonic value: Perceived telepresence (Kim and Biocca 2006; Klein 2003; Nah et al. 2011), Perceived enjoyment (Ghani et al. 1991; Koufaris 2002); Utilitarian value: Perceived product diagnosticity (Jiang and Benbasat 2007), Perceived usefulness (Venkatesh et al. 2017; Vrechopoulos 2004; Xu et al. 2014), Intention to reuse the shopping environment (Carroll and McKendree 1987; Venkatesh et al. 2017; Xu et al. 2014), Perceived ease of use (Davis 1989; Koufaris 2002; Vrechopoulos et al. 2004), NASA task load index (Hart and Staveland 1988), Simulator Sickness (Kennedy et al. 2009)
Pizzi et al. (2019)	Real vs VR	Grocery (Industrial bakery)	Overall satisfaction (Bloemer and de Ruyter 1998), Perceived assortment size (Diehl and Poynor 2018), Hedonic and utilitarian shopping orientation (Babin et al. 1994), Levels of excitement (Wakefield and Baker 1998)
Schnack et al. (2019)	VR vs DVR	Grocery (Miscellaneous)	Telepresence (Witmer and Singer 1998), Usability (Waterlander et al. 2011)
Lombart et al. (2020)	Real vs VR vs DVR	Grocery (Fruit and vegetables)	Appearance and quality (Aurier and Sirieix 2009), Price fairness (Bolton et al. 2018), Perceived healthiness, and hedonism (Bauer et al. 2013), and Consumer attitude (Lombart and Louis 2012)

### Bag shopping experience

For our experimental study, we selected the bag as the product to conduct a case study. We chose this product due to the potential for texture realism and the presence of materials with less complex physics than generic clothes.

In the literature, only three contributions presented a bag shopping experience (Altarteer et al. 2016; Altarteer and Charissis 2019; Wu et al. 2019).

Altarteer et al. (2016) conducted a comparative study to investigate customer attitudes toward a VR system versus a 2D system to customize the products of a luxury brand online. Results demonstrated that the VR system makes available using a high level of product visualization and real-time interaction and promotes hedonic values elevating the customer experience in the shopping environment (Altarteer et al. 2016).

Altarteer and Charissis (2019) presented a VR prototype that enables luxury brand customers to view, interact and customize life-size and photorealistic VR bag models before purchasing. Results indicated that the perceived experience value, presence, ease of use, and usefulness significantly influenced the attitudes toward the VR system (Altarteer and Charissis 2019).

Wu et al. (2019) designed a set of typical VR shopping tasks for the bag shopping experience. Each participant was asked to complete the same shopping task set as quickly as possible using three different interactive techniques: virtual handle controller, raycasting, and user-defined gestures. Results showed that the freehand gesture-based interaction technique was rated as the best in terms of task load, user experience, and presence without the loss of performance (i.e., speed and error count) (Wu et al. 2019).

## Methods

Considering the prior scientific literature and answering the RQs, we performed a comparative study, formulating the following hypotheses:

o H1—The time duration of the shopping experience in IVR is longer than in DVR.
p H2—The proposed IVR-based shopping experience delivers higher hedonic and utilitarian values than DVR.
q H3—Users' cognitive load in IVR does not differ from that in DVR.
r H4—The proposed IVR-based shopping experience gives a better user experience than the DVR-based one.

To test the hypotheses, we performed a within-subjects experiment with two conditions – IVR mode and DVR mode. Both presented the same shopping environment but differed in terms of display systems and interaction. We used a Latin square design to counterbalance the treatment orders by randomly assigning participants to these orders.

### Participants

For this study, 60 participants (36 men and 24 women) ages 22–58 (Mean:30,5 years, SD:10.23) were recruited. Participants included academics and university students from the Polytechnic University of Bari. Most participants (*n *= 53) had at least one to three years of online shopping experience. They had already used VR before the experiment (*n *= 41), with a level of familiarity with this technology equal to 4, measured on a 7-Points Likert scale.

### Task

We designed a virtual fashion shop showcasing gender-fluid clothes and accessories starting from virtual assets purchased online. In the experiment, the task set involved exploring the shop, searching for and selecting a specific bag (shown to users before the experiment), and interacting with the bag and its features.

**Fig. 1:**
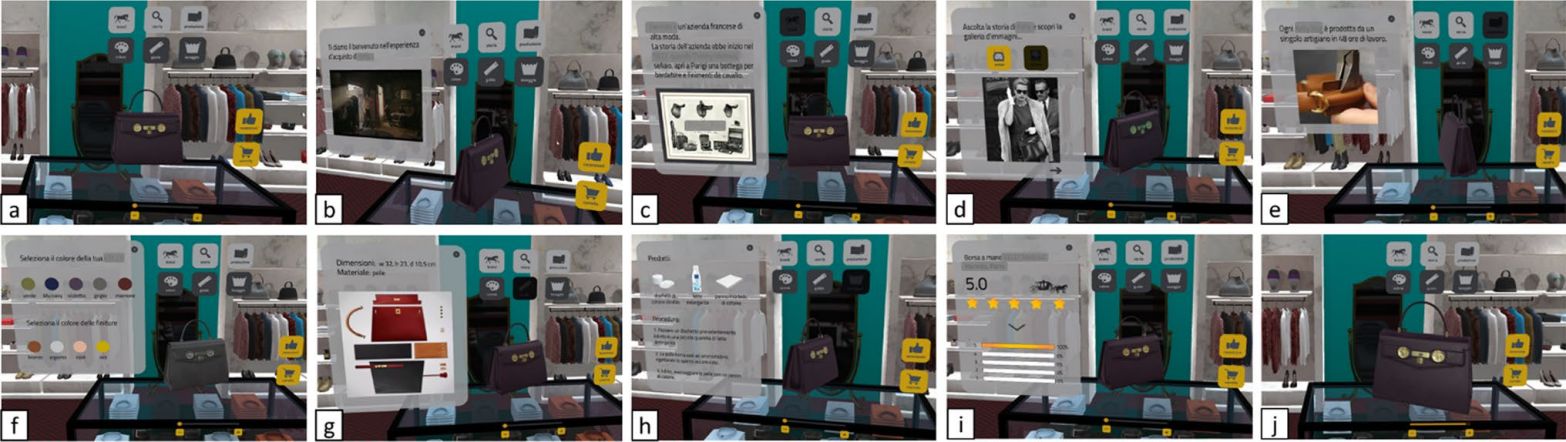
The virtual handbag features within the shopping experience: **a** user interface; **b** presentation trailer; **c** brand; **d** history; **e** production process; **f** color and finishes configuration; **g** size; **h** washing mode; **i** reviews; **j** zoom in

**Fig. 2:**
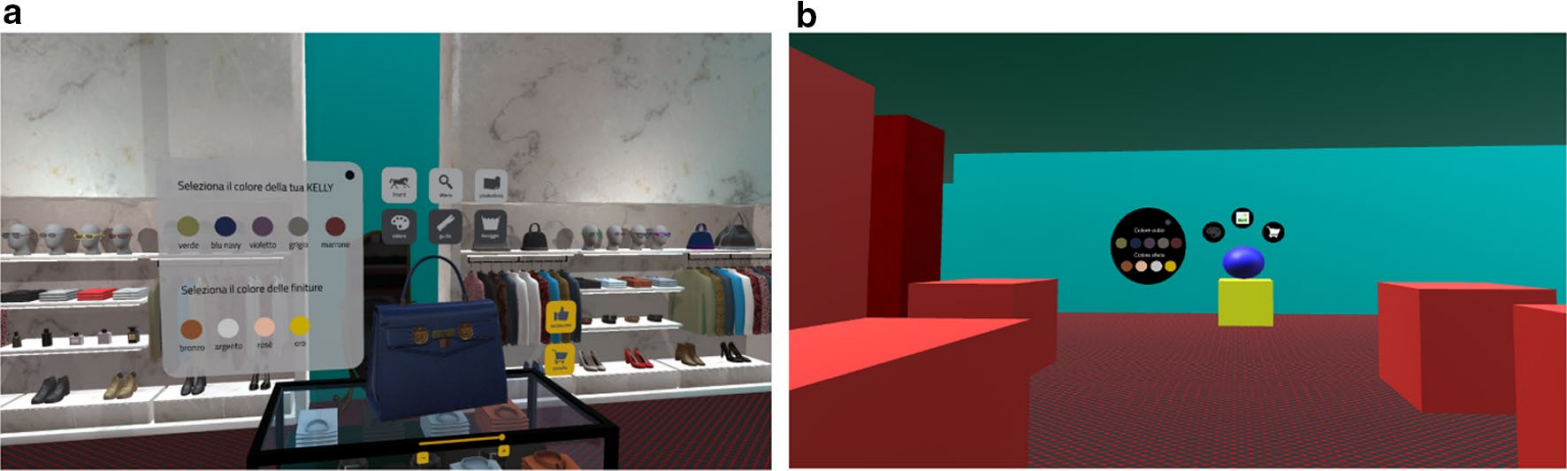
**a** IVR shopping task of the experiment: color and finishes configuration; **b** IVR training scenario with sphere and cube

The bag features included different layers of actions (See Fig. 1):

o Enlarge or shrink the bag.
p View the detailed information for this bag (e.g., brand, history, production process, size, washing mode, and reviews).
q Invoke an attribute window to change its color and its finishes (See Fig. 2a).
r Once finished, put the bag in a shopping cart by clicking on it.

Participants were first instructed on the experimental objective and requirements, followed by an informed consent process. Next, they were introduced to training tasks in DVR and IVR until they completed tasks similar to the real experiment by using the two modes in a training scene with cubes (See Fig. 2b).

The training task concerned:

o Move within the scene.
p Select the red cube.
q Interact with the menu (e.g., invoke an attribute window to change its color and its finishes or a window to see two cube images).
r Click on the shopping cart to complete the experience.

### Experimental setup

We conducted this experiment in the university laboratory. The PC configuration for the experiment consisted of a desktop workstation with an Intel Core i7-10400 processor, 32 GB RAM, and GeForce RTX 3070. The HMD used for the experiment was the Oculus Quest 2 HMD equipped with its two handheld controllers.

**Fig. 3:**
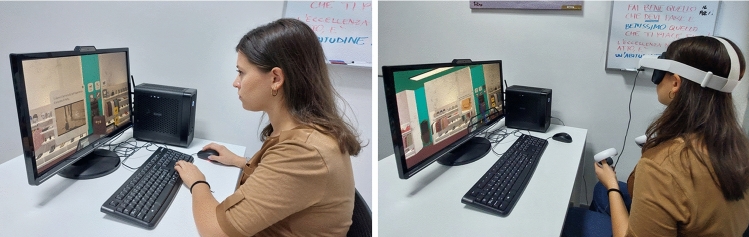
User testing both DVR and IVR versions of the application

To test the differences in terms of metrics, we developed two versions of the application using the Unity engine (See Fig. 3). The first was developed as a traditional desktop application, and the second was developed for Oculus Quest 2. Both versions presented the same functionalities and differed in the interaction and display devices. In the DVR application, interaction occurred with the keyboard and mouse, and the display was on the computer monitor. In contrast, in the IVR application, interaction occurred with the controllers, and the virtual scene was experienced through the HMD. The user was seated during both experiences to avoid sickness in a large shop environment. Locomotion in our IVR scenario was implemented by exploiting the point and teleporting technique (Bozgeyikli et al. 2016), while in the DVR scenario, by pressing the arrow keys on the keyboard.

### Procedure

Upon arrival, participants were greeted by the experimenter and invited to sit at a pre-determined location. The participants were introduced to the purpose and tasks of the experiment through a 5-slide presentation and were invited to provide informed consent. Afterward, we explained to the participants (1) how to interact in both DVR and IVR modes and (2) how to move in both DVR and IVR.

The experimenter administered the pre-experience questionnaire when the participants were ready to start. After completing the pre-experience questionnaire, participants had to experience the training scenes both for DVR and IVR. Once ready, they tested the real shopping scenario to accomplish the task.

Finally, we invited participants to fill out a post-experience questionnaire, both for DVR and IVR modes.

### Measures

We evaluated both objective and subjective measures to test our hypotheses.

#### Objective measures

Concerning hypothesis H1, we compared the time duration of users’ shopping experience in both IVR and DVR modes. We recorded the time duration starting from an event's timestamp (once the experience started in the shop environment) and ending when the user finished the experience (once they clicked on the shopping cart).

#### Subjective measures

We presented three sets of questionnaires to participants: one pre-experiment and two post-experience questionnaires after finishing each session. The questionnaires were designed and distributed to participants using the Google Forms service.

The pre-experience questionnaire consisted of three different sections. The first section included questions about demographic data (i.e., age, gender, nationality, occupation). The second section included questions about the familiarity level with VR and was designed using a 7-Points Likert scale (Albaum 1997). The third section included questions about shopping habits towards online shopping and a final open-ended question where users could suggest how to improve online shopping.

The post-experience questionnaires were also divided into three sections. The first section of the questionnaire concerned the measurement of hedonic and utilitarian values related to hypothesis H2. The questionnaire was based on the model presented by Peukert et al. (2019), and the questions were chosen and modified to fit the scope of this study. The model identified “perceived telepresence and perceived enjoyment as relevant dimensions for the hedonic perspective of the shopping experience and perceived product diagnosticity and perceived usefulness for the utilitarian perspective” (Peukert et al. 2019). All items of the model used a 7-Point Likert scale.

The second section of the questionnaire concerned hypothesis H3. We administered the raw NASA-TLX (RTLX) questionnaire to assess the mental workload (Hart 2016; Hart and Staveland 1988), and we requested that participants fill it out after each mode. We chose the unweighted version of the NASA-TLX because it is easier to administer than the weighted version, and high correlations between the weighted and unweighted scores have been found in the literature (Byers et al. 1989; Moroney et al. 1992).

The third section of the questionnaire was used to assess the user experience. In order to test hypothesis H4, we requested participants to fill out the User Experience Questionnaire (UEQ). The UEQ provided a full depiction of the user experience. Both traditional usability aspects (i.e., efficiency, perspicuity, dependability) and user experience aspects (i.e., novelty, stimulation, attractiveness) were measured (Schrepp et al. 2017) (Table 2).

**Table 2:** Peukert et. al. (2019) model of hedonic and utilitarian values measurement: revised table for the experiment

*Hedonic Value*	
Perceived telepresence	I forgot about my immediate surroundings when I was doing the shopping
	When the shopping task ended, I felt like I came back to the “real world” after a journey
	During the shopping tasks, I forgot that I was in the middle of an experiment
	The shopping environment displayed on the screen (or on the HMD) seemed to be “somewhere I visited” rather than “something I saw.”
Perceived enjoyment	I found my shopping experience interesting
	I found my shopping experience enjoyable
	I found my shopping experience exciting
	I found my shopping experience fun
*Utilitarian Value*	
Perceived product diagnosticity	The shopping environment was helpful for me to evaluate the cloth
	The shopping environment was helpful for me to understand the characteristics of the cloth
	The shopping environment helped familiarize me with the cloth
Perceived usefulness	The shopping environment is useful for doing the shopping
	The shopping environment improves my shopping performance
	The shopping environment enhances my effectiveness when doing the shopping
	The shopping environment increases my shopping productivity

## Results

### Objective measures

#### Time duration of the shopping experience

We compared the time duration of the shopping experience between IVR and DVR modalities by applying the paired sample T-test. In order to check its assumption, we performed the Shapiro-Wilk test of normality to determine whether the paired measurement was normally distributed. As the results indicate that the DVR and IVR samples were not normally distributed, we performed the log transformation (See Table 3). By doing so, the T-test assumptions were met for both the IVR and DVR samples. Considering the verified assumptions of independent observations and normality, we tested the null hypothesis of equality of the means. The T-test allowed us to reject the null hypothesis. On average, the IVR mode performed better (M = 247,11 s) than the DVR mode (M = 179,43 s), and this improvement was statistically significant t (59) =  − 3,811, *p *< 0.05.

**Table 3:** Normality test scores before and after log transformation

	Shaporo-Wilk			Shaporo-Wilk (after log-10 transformation)		
	Statistics	df	Sig	Statistics	df	Sig
DVR	0.914	60	0.000	0.988	60	0.834
VR	0.952	60	0.019	0.984	60	0.619

### Subjective measures

#### Hedonic and utilitarian values

We performed the Shapiro-Wilk test to verify the normality condition for both the hedonic and utilitarian value constructions. Consequently, the DVR and IVR samples were not normally distributed for all constructs (See Tables 4 and 5). Therefore, we performed the Mann-Whitney U test, a technique used to compare differences between two independent groups when the dependent variable is either ordinal or continuous but not normally distributed.

**Table 4:** M, mean; SD, standard deviation. Sig., normality based on Shapiro-Wilk test for the Hedonic Value constructs

	*Hedonic value*					
	Telepresence			Enjoyment		
	M	SD	Sig	M	SD	Sig
DVR	3.62	1.54	0.139	5.35	1.31	0.001
VR	3.55	1.24	0.000	6.41	0.87	0.000

**Table 5:** M, mean; SD, standard deviation. Sig., normality based on Shapiro-Wilk test for the Utilitarian Value constructs

	*Utilitarian value*					
	Product Diagnostlcity			Usefullness		
	M	SD	Sig	M	SD	Sig
DVR	5.28	1.37	0.001	5.29	1.44	0.000
VR	5.95	1.21	0.000	6.08	1.07	0.000

For the telepresence dimension, the p-value is less than 0.001, and the test allowed us to reject the null hypothesis. The mean rank for the telepresence score for the DVR is 40.49, whereas for the IVR is 80.51. This means that the IVR group scores were higher than those in the DVR group. A similar condition occurred for the enjoyment dimension. From the Mann-Whitney U test, the p-value is less than 0.001, and the null hypothesis is rejected. The mean rank for the enjoyment score is 44.39 for DVR, while it is 76.61 for IVR. This means that the scores of the IVR group tend to be higher than those of the DVR group.

**Fig. 4:**
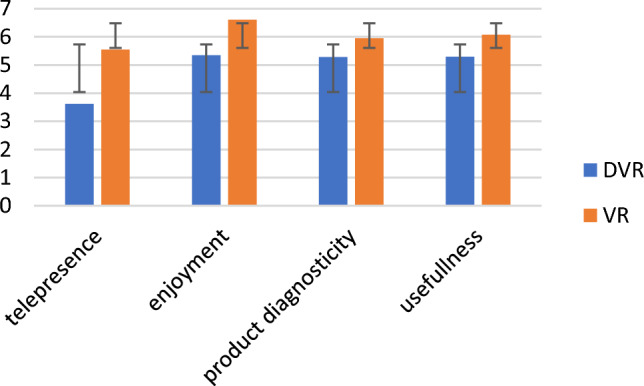
Hedonic and utilitarian dimensions means and standard deviations in IVR and DVR

For both the utilitarian values dimensions, product diagnosticity, and usefulness, the p-value is less than 0.001, and the Mann-Whitney U test allowed us to reject the null hypothesis. The mean rank for the product diagnosticity scores for the DVR is 50.55, whereas for the IVR is 70.45. This means that the scores in the IVR group tend to be higher than those in the DVR group. A similar condition occurred for the enjoyment dimension, where the mean rank score for the DVR is 49.67 while for the IVR is 71.33. Figure 4 reports the comparison between the means and standard deviations obtained by the two modes.

#### Cognitive load (Nasa-RTLX)

We performed the Shapiro-Wilk test to test the normality condition for the cognitive load. As a result, the DVR and IVR samples were not normally distributed (See Table 6). We used a log transformation to distribute the DVR and IVR samples normally (See Table 6).

**Table 6:** Normality test scores before and after log transformation

	Sharpiro-Wilk			Sharpiro-Wilk (after log-10 transformation)		
	Statistics	df	Sig	Statistics	df	Sig
DVR	0.883	60	0.000	0.988	56	0.066
VR	0.943	60	0.008	0.984	56	0.095

**Fig. 5:**
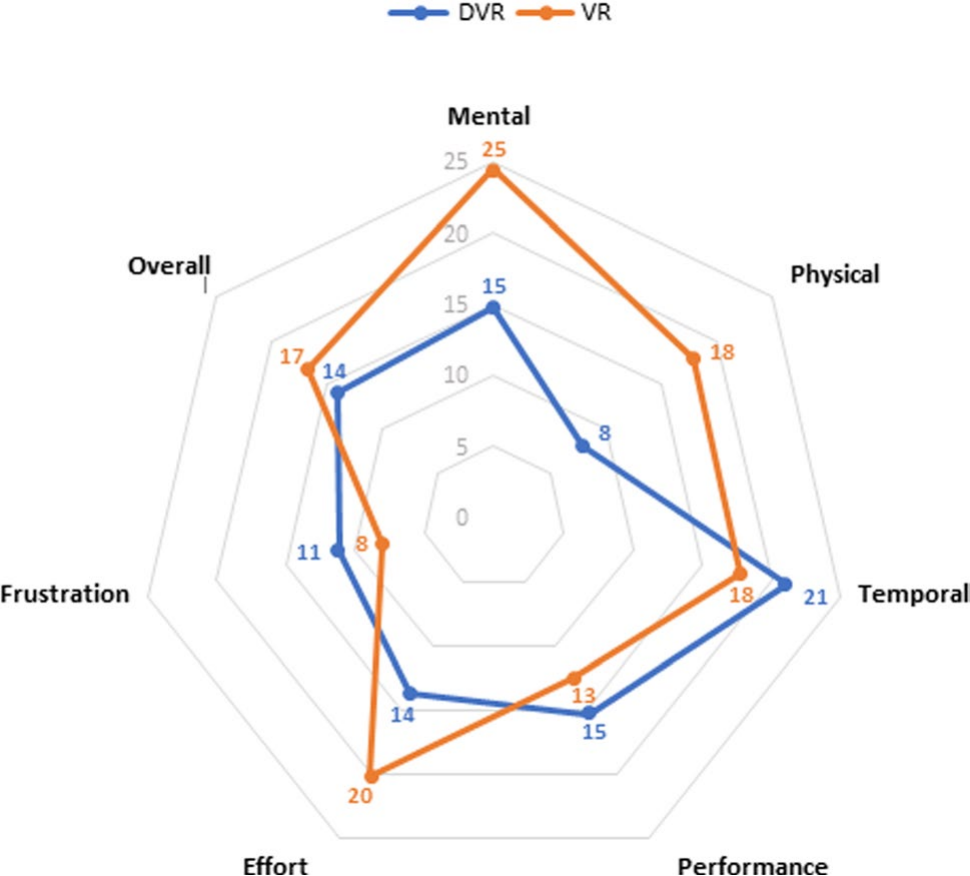
Comparison of the overall and the Nasa-RTLX subscales of IVR and DVR modes

As the T-student assumptions were fulfilled, we used the paired-samples T-test to compare the RTLX results. The mean value of the overall RTLX score for the IVR mode was comparable to the DVR mode. The T-test did not allow us to reject the null hypothesis. Thus, the difference was not statistically significant (17 vs. 14, t (55) =  − 1,854, *p *> 0.069, See Fig. 5).

#### User experience

The User Experience Questionnaire (UEQ) is a commonly used questionnaire for measuring consumers' subjective opinions about the user experience of products (Laugwitz et al. 2008). The UEQ is a semantic differential with 26 items that are made of adjectives. The purpose of UEQ is to understand how users consider the system by six dimensions: attractiveness, perspicuity, efficiency, dependency, stimulation, and novelty. The UEQ scores concerning the six scales, together with the corresponding Cronbachs-Alpha coefficients, are listed in Table 7. The Alpha coefficient (Cronbach 1951) is a measure of a scale's consistency. Unfortunately, there is no universally acknowledged rule for determining the size of the coefficient. To be deemed adequately consistent, a scale should have an Alpha value greater than 0.7 (Schrepp 2019). This rule is verified for five scales, except for one, *dependability*, which shows in both IVR mode and DVR mode an Alpha < 0.7(*). This may be an indication that *dependability* items are interpreted unexpectedly by different participants.

**Table 7:** UEQ Scores with dependability inconsistencies

	VR mode		DVR mode	
	Mean	Cronbachs Alpha	Mean	Cronbachs Alpha
Attractiveness	2.35	0.88	1.84	0.91
Perspicuity	2.42	0.75	2.05	0.84
Efficiancy	2.26	0.75	1.87	0.80
Dependability	1.56	0.37*	1.62	0.40*
Stimulation	2.38	0.83	1.79	0.89
Novelty	2.47	0.76	1.79	0.89

*Alpha < 0.7

This is certainly due to the inconsistent answers on the scale. In fact, the *dependability* in the IVR mode presents 25 items with a Critical Indicator (CI) equal to 2, and the DVR mode presents 18 items with CI = 2. This can also result from random response errors or misunderstanding of an item. In our case, it does not make sense to consider dependability as a problem because this only occurs for a single scale (Schrepp 2019).

**Fig. 6:**
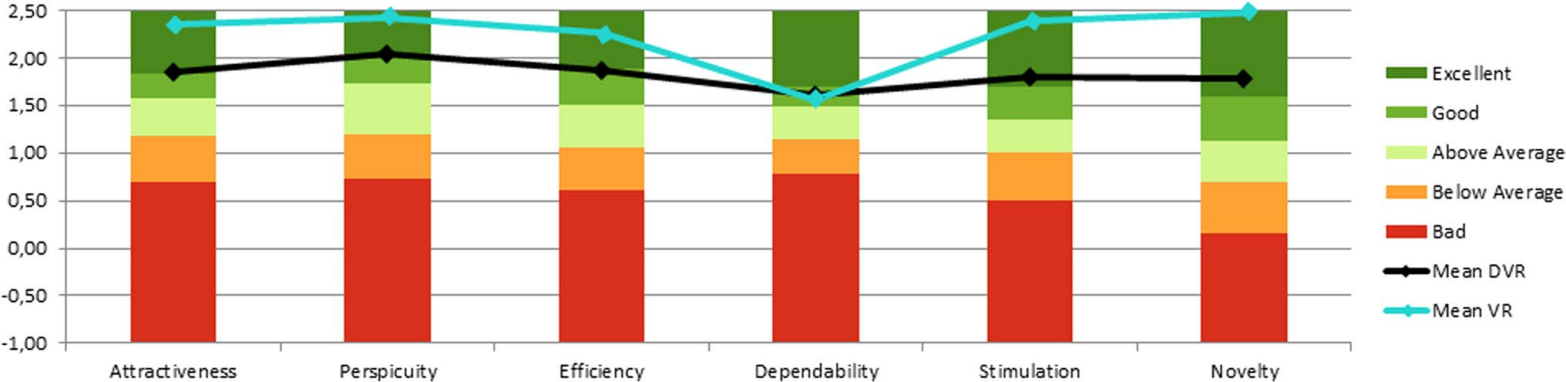
The UEQ benchmark histogram comparison of IVR mode against DVR mode

We used the UEQ analysis tool (Schrepp et al., 2017) to compare the two UEQ datasets using a data collection containing data from 21,175 people from 468 studies on various items as a baseline. Figure 6 reports the comparison between the scores obtained by the two modes.

## Discussion

The comparison of experience duration times confirms our H1 hypothesis. The longer duration of the experience in IVR is related to user feelings of higher immersion and engagement in VR than in DVR due to a renewed sense of novelty (Huang et al. 2021). In fact, although one-third of the sample (*n *= 19) had never used IVR before, immersion times in the virtual experience are significantly higher than in DVR (M_VR_ = 247.11 s vs. M_DVR_ = 179.43 s). User feedback collected through observation also confirms their willingness to remain immersed in the IVR shop and a few (*n *= 11) wished they had not clicked the shopping cart to complete the experience.

Regarding the statistically significant difference between hedonic and utilitarian values and their constructs, the results show that IVR has higher hedonic and utilitarianism than DVR, confirming hypothesis H2. Indeed, two-dimensional shopping experiences are insufficient for delivering a high hedonic purchasing experience (Goldsmith and Flynn 2005). Furthermore, hedonic shopping experiences involving positive emotions have been connected to various crucial outcomes, such as greater time spent in the store, validating the H1-H2 correlation (Babin et al. 1994; Jones et al. 2006). As a result, IVR can be considered a valuable technology for increasing consumers' hedonic shopping experiences. Results provide initial evidence supporting the existence of both utilitarian and hedonic shopping orientations in IVR-based stores than DVR (Pizzi et al. 2019).

Hypothesis H3 is confirmed by the cognitive load assessment. The non-significance of the results is supported by an Alpha Cronbachs coefficient lower than 0.7. Although preliminary results show that cognitive load in both versions is comparable, further study should be carried out to assess whether frustration and temporal demand are statistically significant. Frustration should be higher in the DVR version, confirming that, unnatural interaction techniques, such as DVR mode could increase users' cognitive load (e. g., frustration) (Wu et al. 2019). Also, there should be a difference in temporal demand showing that users perceive IVR shopping experience faster than DVR. This could be connected to the higher degree of immersion that engages users (H1).

Also, Hypothesis 4 is confirmed, showing that there is a difference between user experience rates in IVR and DVR. In fact, IVR delivers better results in terms of attractiveness (Peukert et al. 2019), perspicuity, efficiency, stimulation, and novelty. For dependability, there should have been some misunderstandings about items confirmed in the IVR mode by 25 items with a Critical Indicator (CI) equal to 2, and in the DVR mode by 18 items with CI = 2.

We, therefore, believe that perceived hedonism and utilitarianism may depend on how the IVR system is implemented compared to the DVR system. We hypothesize that the explanation lies in the display and interaction systems and the relative degree of immersiveness.

In the case of the DVR application, the display system was on a 2D screen with a low degree of immersiveness. In the case of the IVR, on the other hand, users could be fully immersed in a virtual environment surrounding at 360°. Recent literature reports that IVR displays have been shown to positively influence performance in a visual search task (Pallavicini and Pepe 2019), such as the bag search in our experiment.

Moreover, we consider that the high values of hedonism and utilitarianism could also be due to the HMD used for the experiment, i.e. the Oculus Quest 2, which had a high resolution compared to the HTC Vive used by Peukert et al. (2019) for their experiment 5 years earlier. Indeed, the state of technology may condition generalizability to future computing artifacts because of the dependence of the results on current technology.

In addition, users feel more naturally immersed in the IVR scenario, showing a more intense emotional response in IVR than DVR (Othman et al. 2022). Moreover, users commented that they felt a greater sense of presence in the IVR condition than in the DVR condition, probably because of the greater degree of immersiveness due to the HMD stereoscopic view.

Indeed, interaction systems could also be contributing factors. For instance, in IVR we used controllers to implement the virtual-hands technique (Argelaguet et al. 2016) and the raycasting-based virtual pointer (Lee et al. 2003).

The virtual hands are 3D models that correctly represent human hands in terms of size and appearance and offer an isomorphic mapping between the user's actual and virtual hands. In order to reach far-off targets and engage more fluidly with UI elements, we have also designed a laser-pointer metaphor that is based on the Raycasting technique. Raycasting is a series of interactive techniques used in IVR for distant target selection. We developed a laser beam to represent a Raycasting-based virtual pointer, with the controller acting as the input device with six degrees of freedom. It is possible to choose a target object when the laser beam crosses it. In contrast, in DVR the interaction was the mouse-based point selection.

Therefore, our findings indicate that there is a correlation between the degree of immersiveness of the technology under consideration and the hedonic and utilitarian values of the shopping experience, as anticipated by Childers et al. (2001) for "new media".

Moreover, as Peukert et al. (2019) stated, the effect of immersion on hedonic and utilitarian values should be more pronounced (positive) for high-involvement products, such as fashion products, as opposed to low-involvement products, such as grocery products.”

We, therefore, designed a table (See Table 8) to provide future researchers an overview of the DVR and IVR shopping experiences in previous comparative studies and in our own study. Our focus was about the interaction, visualization, and locomotion systems implemented, the related products compared (low–high involvement), and the results with respect to hedonic and utilitarian value.

**Table 8:** General framework of DVR-IVR studies related to hedonism and utilitarism

Shopping experience modes	Display	Interaction	Locomotion	Hedonism	Utilitarism
DVR (high involvement product)	Desktop computer screen	Mouse cursor	Keyboard arrow keys	Low	Low
IVR (high involvement product)	IVR headset (Oculus Quest 2)	Raycasting laser pointer interaction	Teleport metaphor	High	High
DVR (low involvement product)	Desktop computer screen	Keyboard and a mouse (Lombart et al. 2020) Mouse cursor (Peukert et al. 2019)	Keyboard arrow keys and mouse (Lombart et al. 2020) Keyboard arrow keys (Peukert et al. 2019)	Low (Peukert et al. 2019) High (Lombart et al. 2020)	High (Peukert et al. 2019)
IVR (low involvement product)	Oculus Rift DK2 (Lombart et al. 2020) HTC Vive (Peukert et al. 2019)	Raycasting gaze pointer interaction (Lombart et al. 2020) Virtual handle controller (Peukert et al. 2019)	Game controller with two thumbsticks (Lombart et al. 2020) Natural walking (Peukert et al. 2019)	High (Lombart et al. 2020; Peukert et al. 2019)	Low (Peukert et al. 2019)

Our aim is to provide a comprehensive framework of shopping experiences that could be useful for researchers who want to undertake similar comparative studies in other retail areas as well.

This study, however, has some drawbacks. First, it involves only one task related to the shopping experience of a bag. Therefore, we did not investigate the effectiveness and contribution of other fashion products (e.g., clothes). In addition, some users stated that the shopping experience would probably be more convenient if performed while standing rather than sitting. Therefore, future studies could use smaller shop environments in order to avoid cybersickness through room-scale experiments. In addition, future research may also include the development of virtual mirrors within which users can mirror themselves with the purchased product in order to increase their presence (Witmer and Singer 1998). Finally, we tested the application in IVR and DVR but not in a real scenario, as in other comparative studies (in the grocery sector) (Bressoud 2013; Lombart and Louis 2012; Pizzi et al. 2019; van Herpen et al. 2016; Waterlander et al. 2015). In this way, we will also be able to evaluate other interesting aspects in addition to the metrics already used such as purchase intention, % of items purchased, and customer engagement.

## Conclusion

In the age of e-commerce, IVR represents a powerful tool and an opportunity for enhancing shopping activities, particularly for the fashion industry. To this end, the “virtuality” of the proposed shopping experiences can be integrated into e-commerce in both immersive and non-immersive ways.

The existing literature provides only comparative studies in another retail sector (e.g., groceries). Whereas this paper proposes the first comparative study between IVR and DVR in the fashion industry concerning a bag shopping experience. The results show that the experience in IVR presents better results in terms of hedonic and utilitarian value and user experience than in DVR. The cognitive load in both modes is comparable and the experience duration time is higher in IVR than in DVR. Although our study only considers one possible implementation of the shopping experience with respect to a bag, the findings support the use of IVR technologies for shopping and pave the way for future research in the fashion industry.

In the future, we intend to extend our research by evaluating the effectiveness of IVR to improve the online shopping experience by contributing to a more sustainable fashion industry. IVR could help reduce the environmental impact of apparel production. In fact, by configuring and displaying apparel in IVR before purchase, companies can take advantage of online shopping by starting the actual production of clothes and accessories only once they are placed in the shopping cart, without needing to produce them prior and stock them in their inventories. We also plan to conduct a user study in a real shopping scenario to enrich the three-axis comparative study (IVR, DVR, real) and collect users' feedback regarding the three shopping modes.

## Supplementary Information

Below is the link to the electronic supplementary material.

Supplementary file1 (MP4 158305 KB)

## Data Availability

Data will be made available on reasonable request.
